# Matrix-Assisted Laser Desorption Ionization Time-of-Flight Mass Spectrometry Combined with Chemometrics for Protein Profiling and Classification of Boiled and Extruded Quinoa from Conventional and Organic Crops

**DOI:** 10.3390/foods13121906

**Published:** 2024-06-17

**Authors:** Rocío Galindo-Luján, Laura Pont, Fredy Quispe, Victoria Sanz-Nebot, Fernando Benavente

**Affiliations:** 1 Department of Chemical Engineering and Analytical Chemistry, Institute for Research on Nutrition and Food Safety (INSA·UB), University of Barcelona, 08028 Barcelona, Spain; rgalindo@ub.edu (R.G.-L.); laura.pont@ub.edu (L.P.); vsanz@ub.edu (V.S.-N.); 2 Serra Húnter Program, Generalitat de Catalunya, 08007 Barcelona, Spain; 3 National Institute of Agricultural Innovation (INIA), Lima 15024, Peru; equispe@inia.gob.pe

**Keywords:** boiling, conventional farming, extrusion, MALDIquant, MALDI-TOF-MS, multivariate data analysis, organic farming, proteins, quinoa

## Abstract

Quinoa is an Andean crop that stands out as a high-quality protein-rich and gluten-free food. However, its increasing popularity exposes quinoa products to the potential risk of adulteration with cheaper cereals. Consequently, there is a need for novel methodologies to accurately characterize the composition of quinoa, which is influenced not only by the variety type but also by the farming and processing conditions. In this study, we present a rapid and straightforward method based on matrix-assisted laser desorption ionization time-of-flight mass spectrometry (MALDI-TOF-MS) to generate global fingerprints of quinoa proteins from white quinoa varieties, which were cultivated under conventional and organic farming and processed through boiling and extrusion. The mass spectra of the different protein extracts were processed using the MALDIquant software (version 1.19.3), detecting 49 proteins (with 31 tentatively identified). Intensity values from these proteins were then considered protein fingerprints for multivariate data analysis. Our results revealed reliable partial least squares-discriminant analysis (PLS-DA) classification models for distinguishing between farming and processing conditions, and the detected proteins that were critical for differentiation. They confirm the effectiveness of tracing the agricultural origins and technological treatments of quinoa grains through protein fingerprinting by MALDI-TOF-MS and chemometrics. This untargeted approach offers promising applications in food control and the food-processing industry.

## 1. Introduction

Quinoa (*Chenopodium quinoa* Willd.) is an important crop originally from the Andes Mountains in Peru, Bolivia, and Chile. This “Golden Grain” is in global demand for its exceptional nutritional and immuno-nutritional properties [1,2,3,4]. Quinoa is a rich source of gluten-free proteins containing all essential amino acids, important minerals, omega-3 fatty acids, polyphenols, and vitamins, along with other interesting bioactive compounds [3,5]. Among these compounds, saponins exhibit haemolytic activity and induce bitterness. However, they are effectively removed from the seeds using various methods, such as washing and abrasion [6]. Quinoa is resilient to environmental stress and poor soil, making cultivation a viable option worldwide [3,7,8]. In recent years, the cultivation of organic quinoa has experienced a dramatic increase, because it is perceived as safer, healthier, and more environmentally friendly than quinoa from conventional farming [9,10,11]. White quinoa, known for its high productivity, is the most widely cultivated commercial variety [12].

The extensive exploration of technological approaches has been undertaken to improve the nutritional and functional potential of quinoa-based products, aiming to enhance the potential benefits of incorporating quinoa into the diet. The typical technological methods described in the literature can be classified based on whether they involve heat energy input during processing. Thermal treatment methods typically include extrusion, drying, and boiling, under or without pressure. Conversely, nonthermal treatment methods involve high hydrostatic pressure, atmospheric pressure, cold plasma, and sonication [2]. Among thermal treatment methods, extrusion is considered a versatile and efficient technique for processing instant foods with diverse textures and shapes. This process involves the application of heat, mechanical energy, and pressure. During extrusion, the starch in quinoa seeds undergoes gelatinization, and the proteins denature, improving digestibility. However, it is noteworthy that the protein and lipid content in extruded quinoa typically diminishes due to the formation of protein–lipid or starch–lipids complexes, resulting in a remarkable decrease in solubility [2,13]. On the other hand, boiling can also enhance digestibility and bioavailability while promoting sensory properties like palatability, taste, flavour, and the development of soft and mushy textures. However, cooking also affects the composition of numerous chemical constituents, including proteins, amino acids, vitamins, and minerals [14]. 

Research on the impact of agricultural production methods and technological treatments on quinoa proteins remains very limited [15,16,17,18,19]. These studies are a necessary part of the quality control of quinoa grains and their derived products, which face the threat of adulteration with cheaper cereals [20,21,22,23]. Food adulteration is a widespread malpractice aimed at maximizing economic benefits, posing potential risks to human health by either depriving consumers of vital nutrients or exposing them to allergenic or toxic compounds [20,24,25,26]. Consequently, there is an urgent need to develop analytical methods for quinoa characterization aimed at enhancing quality control, food-safety, and fraud-prevention programs.

Different analytical techniques assisted by chemometrics for data deconvolution, multivariate data analysis, and classification have been described for the characterization of quinoa [20,21,22,23,27,28,29,30,31,32]. Several authors have demonstrated the potential of using infrared or fluorescence spectroscopic techniques to obtain global profiles of quinoa flour components for tracing adulteration [20,21,22,23]. Other authors have targeted the volatile fraction of compounds in quinoa flour for the same purpose, using headspace–gas chromatography–ion mobility spectrometry (HS-GC-IMS) [27]. Alternatively, we have been focused on the global profiling of quinoa proteins, which has proven to be an efficient way to characterize commercial varieties of quinoa grains [28,29,30,31]. We have developed different methods based on capillary electrophoresis and liquid chromatography with ultraviolet absorption spectrophotometric detection (CE-UV and LC-UV, respectively) [28,29], shotgun proteomics using label-free liquid chromatography coupled to tandem mass spectrometry (LC-MS/MS) [30], and matrix-assisted laser desorption ionization time-of-flight mass spectrometry (MALDI-TOF-MS) [31]. In particular, the MALDI-TOF-MS method proved to be highly convenient, enabling the rapid, straightforward, and reliable differentiation of commercial quinoa grains based on the proteins detected in their characteristic mass spectra [31]. Additionally, the most relevant proteins for discriminating between different quinoa grains were tentatively identified based on their molecular masses (M_r_), comparing them with the experimental proteome map obtained by LC-MS/MS shotgun proteomics [30]. Protein identification not only enhances the reliability of differentiation but also provides valuable information, such as the potential bioactivity of the present proteins [32].

In this study, we extend the previously developed MALDI-TOF-MS global profiling approach to discriminate among commercial quinoa grain varieties, aiming to investigate the impact of agricultural production methods and technological treatments on quinoa proteins. We employ MALDI-TOF-MS to obtain global profiles of quinoa proteins from white quinoa varieties cultivated under two distinct farming practices (organic and conventional) and subjected to different processing methods (boiling and extrusion). Subsequently, MALDIquant and chemometrics are applied for efficient data processing and multivariate analysis. Additionally, the method tentatively identifies the most critical proteins for discrimination within the analysed samples, providing insights that may have important nutritional, functional, and technological implications. Ultimately, this information can contribute to the improvement of agricultural and food production practices.

## 2. Materials and Methods

### 2.1. Chemicals

All the chemicals were of at least analytical reagent grade. Hydrochloric acid (37% (*v*/*v*)), sodium hydroxide (≥99.0%, pellets), boric acid (≥99.5%), water (LC-MS grade), acetonitrile (ACN, LC-MS grade), acetone (99.8%), sinapinic acid (SA, ≥99.0%), and trifluoroacetic acid (TFA, 99.0%) were provided by Merck (Darmstadt, Germany). Milli-Q ultra-pure water system (Millipore, Molsheim, France) was employed for water purification.

### 2.2. Samples

The investigation involved triplicate analysis of four distinct white quinoa variety samples, including raw quinoa (seeds and grains), two crop conditions (conventional and organic), and two processing conditions (boiling and extrusion). The quinoa varieties, namely Quillahuaman INIA (Quillahuaman, V1), INIA 433-Santa Ana/AIQ/FAO (Santa Ana, V2), INIA 431-Altiplano (Altiplano, V3), and Salcedo INIA (Salcedo, V4), were provided by the National Institute of Agrarian Innovation (INIA) from Lima, Peru. These four quinoa varieties were cultivated in both conventional and organic conditions in La Molina (Lima, Peru) (latitude 12°04′36″ S, longitude 76° 56′43″ W, altitude 241 m above sea level (masl)) and Omas (Lima, Peru) (latitude 12°33′25.6″ S, longitude 76°19′9″ W, altitude 1227 masl), respectively. They were grown in the same year (2018) to minimize environmental effects. 

Conventional soil fertilization was performed using a mixture containing urea, potassium chloride, and diammonium phosphate, while organic soil fertilization employed ‘Bokashi’, a fermented food-based fertilizer comprising organic materials such as animal dung, yeast, and molasses. Quinoa seeds were processed using a scarifier machine (Vulcano, Lima, Peru) to separate the grain from the pericarp. To eliminate saponins responsible for the bitter flavour, the obtained quinoa grains were washed three times for 5 min in a quinoa-to-water 1:10 (*m*/*v*) bath at room temperature (rt). Finally, the washed quinoa grains were dried at 40 °C in an oven (Memmert, Schwabach, Germany) and stored in a dry environment at rt. 

### 2.3. Extrusion Process 

White quinoa grains from the four varieties, cultivated under both conventional and organic farming methods, were preconditioned with water (12–14% moisture) to achieve optimal heat transfer during the extrusion process and ensure starch gelatinization. Extrusion took place in a co-rotating twin-screw extruder (Inbramaq, São Paulo, Brazil) with a total barrel length of 960 mm, a screw diameter of 30 mm, and a cylindrical die diameter of 10 mm. The extruder featured three independent zones: a feeding zone, a heating zone, and a die zone. Temperature settings were as follows: the feeding zone was maintained at 30 °C, gradually increasing to 40 °C and then 50 °C. The heating zone had variable temperatures of 70 °C, 85 °C, and 100 °C, while the die zone was set at temperatures of 100 °C, 110 °C, and 125 °C. The grain feed rate was established at 14 kg/h, with a screw speed of 800 rpm. The cut-off frequency was configured at 17 Hz, keeping the retention time between 10 and 15 s. After the extrusion process, the extruded grains were cooled for 15 min and subsequently stored in polyethylene (PE) bags at rt until further analysis.

### 2.4. Boiling Process

Another batch of white quinoa grain samples was milled utilizing a laboratory ultra-centrifugal mill (Restch, Schwabach, Germany) at 18,000 rpm for 30 s. The milling process involved sieving through a mesh with a 0.5 mm opening. The resulting sieved flour was dispersed in water before boiling to prevent lump formation, ensuring a homogeneous mixture. This mixture was then boiled in a cooking pot at 100 °C for 20 min, maintaining a flour-to-water mixture ratio of 1:20 (*m*/*v*) with continuous stirring. Finally, the boiled quinoa was cooled for 20 min, dried at 40 °C for 72 h, and subsequently stored in PE bags at rt until further analysis.

### 2.5. Sample Preparation

Protein extraction from raw (i.e., seeds and grains), boiled, and extruded quinoa from conventional and organic farming was carried out in triplicate for each variety (V1, V2, V3, and V4), resulting in a total of 96 quinoa protein extracts. The extraction protocol was as described in our previous work [30], with some modifications. Briefly, 250 mg of each sample was mixed with 2 mL of water and 39 µL of 1 M NaOH (final pH of 10.0) using a vortex Genius 3 (Ika^®^, Staufen, Germany) for 3 h at rt. The resulting suspension was centrifuged at 23,000× *g* for 60 min at 4 °C in a cooled Rotanta 460 centrifuge (Hettich Zentrifugen, Tuttlingen, Germany). The supernatant was collected, and the pH value was adjusted to 5.0 with 22 µL of 1 M HCl. After centrifugation at 30,000× *g* for 30 min at 4 °C, precipitated proteins were resuspended in 1 mL of a solution of 60 mM H_3_BO_3_ (pH adjusted to 9.0 with NaOH). The resulting solution was filtered through 0.22 µm nylon filters (MSI, Westboro, MA, USA) before analysis. All pH measurements were made using a Crison 2002 potentiometer and a Crison electrode 52-03 (Crison Instruments, Barcelona, Spain).

The estimation of protein content in the quinoa extracts was determined spectrophotometrically utilizing a capillary electrophoresis (CE) instrument equipped with a diode array detector (7100 CE, Agilent Technologies, Waldbronn, Germany). Three independent replicates of samples, obtained from seed, grain, boiled, and extruded quinoa from conventional and organic farming, were injected at 50 mbar for 10 s in a fused silica capillary of 58 cm total length (L_T_), 50 μm internal diameter (i.d.), and 365 μm outer diameter (o.d.) (Polymicro Technologies, Phoenix, AZ, USA). A calibration curve was established using BSA standard solutions at 100 to 1000 mg·L^−1^. Flow injection experiments were performed without voltage, with the sample plug mobilized through applications of 50 mbar pressure after the injection. Absorbance measurements were taken at 214 nm within the region of the detected protein peaks.

### 2.6. MALDI-TOF-MS

For the preparation of the protein extracts for MALDI-TOF-MS analyses, MF-Millipore^®^ membrane filters (Merck) and Milli-Q water were employed for desalting [31]. Briefly, 10 µL of protein extracts were deposited onto the membrane filter, and desalting was achieved by dialyzing with water for 45 min at rt. The dialyzed extracts were then collected and stored at −20 °C until the analyses. 

A 4800 MALDI TOF/TOF mass spectrometer (Applied Biosystems, Waltham, MA, USA) was employed to acquire mass spectra in mid-mass positive mode within a 3000–25,000 *m*/*z* range. Data acquisition and processing were conducted using the 4000 Series Explorer^TM^ (Applied Biosystems, version 3.5) and Data Explorer^®^ (Applied Biosystems, version 4.5) software. Sample-MALDI matrix mixtures were freshly prepared as described in our previous work [31]. Briefly, the procedure involved manually spotting, droplet-by-droplet, onto a steel MALDI plate 1 μL of a 27 mg·mL^−1^ SA solution in 99:1 (*v*/*v*) acetone:water, 1 µL of dialyzed sample solution, an additional 1 µL of dialyzed sample solution (for enhanced homogeneity), and finally 1 µL of a 10 mg·mL^−1^ SA solution in 50:50 (*v*/*v*) ACN:water with 0.1% (*v*/*v*) of TFA. Between each droplet addition, spots were allowed to dry at rt. The resulting layer-by-layer spots ensured maximal homogeneity and reproducibility in the MALDI-TOF-MS analyses. Each of the 96 quinoa protein extracts was spotted and analysed in triplicate.

### 2.7. Data Analysis

The MALDI-TOF mass spectra were processed and analysed employing MALDIquant and multivariate data analysis [31]. 

#### 2.7.1. MALDIquant Data Processing

The raw mass spectra were initially converted to text format (.txt) using Data Explorer^®^ software (version 4.5, accessed on 1 December 2023). Afterward, the raw mass spectra were imported into the R platform (version 4.0.4, http://www.R-project.org/, accessed on 1 January 2024) [33] with the MALDIquantForeign package (version 0.12) [34]. MALDIquant (version 1.19.3) [35] was then employed to detect protein peaks in the mass spectra based on their characteristic *m*/*z* and intensity values. Imported data from the 96 protein extracts (3 spots c/u) were first transformed for variance stabilization through a square root transformation [36]. Smoothing was applied to enhance the signal-to-noise ratio (SNR) and reduce noise in the mass spectra using the Savitsky–Golay algorithm filter in profile mode [37]. Subsequently, the baseline was subtracted using the sensitive nonlinear iterative peak (SNIP) algorithm [38]. The denoised data were then normalized, setting the total ion current to one [39]. After that, alignment was achieved using the warping algorithm facilitated by locally weighted scatterplot smoothing (LOWESS) [40]. Following alignment, the mass spectra from replicates were averaged to derive a mean mass spectrum for each of the 96 protein extracts. Then, a peak detection algorithm based on the median absolute deviation (MAD) was applied to detect features of potential proteins [41]. Finally, a peak binning procedure, using the binpeaks function, was implemented to compensate for small variations in the *m*/*z* values. 

#### 2.7.2. Multivariate Data Analysis

Multivariate data analysis was conducted using the PLS Toolbox (Version 9.0, Eigenvector Research Incorporated, Wenatchee, WA, USA) in Matlab R2016a (The MathWorks Incorporated, Natick, MA, USA). Principal component analysis (PCA) and partial least squares discriminant analysis (PLS-DA) were performed using the scaled intensities of the proteins detected with MALDIquant. PCA served for the unsupervised assessment of general clustering trends among different farming and treatment conditions, as well as for detecting potential outliers. Subsequently, PLS-DA was used to maximize the separation between observed sample classes, constructing a classification model [42,43]. For model optimization, a leave-one-out cross-validation model was performed [44]. Membership within each class was examined within a 95% confidence ellipse in the PLS-DA score plot [45]. Variable importance in the projection (VIP) scores [44,46] was also calculated to investigate the degree of influence of each individual protein on discrimination. Finally, the most relevant proteins for discriminating between the sample classes were tentatively identified based on their M_r_, comparing them with the experimental proteome map of the Salcedo white quinoa grains obtained in a separate study by LC-MS/MS shotgun proteomics [47].

## 3. Results and Discussion

### 3.1. MALDI-TOF-MS Analysis

**Figure 1:**
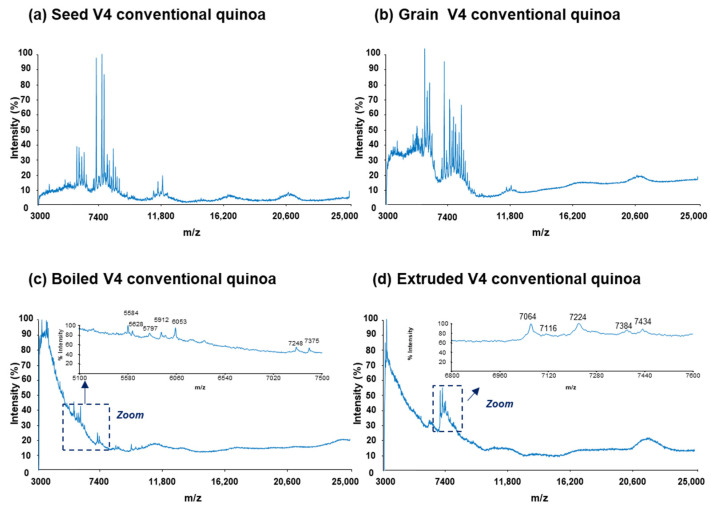
Raw MALDI-TOF mass spectra for the protein extracts of (**a**) seed, (**b**) grain, (**c**) boiled, and (**d**) extruded V4 quinoa varieties from conventional farming (V4 = Salcedo). Different regions of the mass spectra are zoomed in for boiled and extruded quinoa.

**Figure 2:**
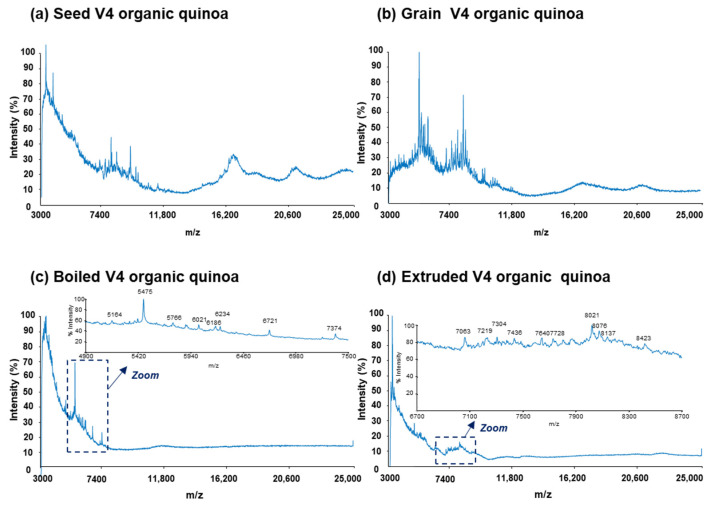
Raw MALDI-TOF mass spectra for the protein extracts of (**a**) seed, (**b**) grain, (**c**) boiled, and (**d**) extruded V4 quinoa varieties from organic farming (V4 = Salcedo). Different regions of the mass spectra are zoomed in for boiled and extruded quinoa.

To obtain characteristic mass spectra profiles of protein extracts from quinoa, we employed a reliable and reproducible sample preparation method described in our previous study [31]. This sandwich method, previously used on raw commercial quinoa grains, was applied to the preparation of sample-MALDI matrix mixtures and spot deposition. Figure 1 and Figure 2 present representative mass spectra for the protein extracts of seed, grain, boiled, and extruded V4 quinoa varieties from conventional and organic farming, respectively. 

In particular, Figure 1 and Figure 2a,b display the mass spectra of the protein extracts from seeds and grains under both farming conditions. As can be observed, characteristic mass spectra rich in proteins were obtained within the scanned range of 3000 to 25,000 *m*/*z* in all cases. Moreover, the differences in mass spectra were more pronounced when comparing seeds and grains for a specific farming type. This could be attributed to the technological treatments applied to quinoa seeds to prepare grains, including scarification and, particularly, washing and drying, aimed at reducing the high levels of saponins.

On the other hand, Figure 1 and Figure 2c,d illustrate the mass spectra of the protein extracts from boiled and extruded quinoa under both farming conditions. As can be observed, both boiling and extrusion treatments led to a reduction in the detected proteins. Indeed, the total amount of protein in these processed quinoa extracts was lower compared to the raw quinoa (i.e., seeds and grains), with, for example, 1.1% and 5.5% (*m*/*m*) for extruded and seed V4 quinoa varieties from conventional farming, respectively. Such behaviour can be attributed to the effect of heat and pressure treatments on the reduction in protein solubility, resulting in protein denaturation, oxidation, and aggregation [14,48,49,50].

To generate a reliable large data set for multivariate data analysis, mass spectra were collected in triplicate (*n* = 288 spots (96 × 3)) for all the protein extracts of seed, grain, boiled, and extruded quinoa samples from the four varieties grown under both conventional and organic farming methods. However, direct peak detection for protein fingerprinting was challenging due to the complexity of the mass spectra, which exhibited numerous overlapped protein peaks with varying intensities. Consequently, we employed MALDIquant software (version 1.19.3) for the efficient quantitative processing of the mass spectra, as described in our previous work [31]. This approach facilitated improved peak detection, reliably providing distinctive protein features with characteristic *m*/*z* and intensities. Such accuracy was essential for subsequent multivariate data analysis by PCA and PLS-DA to discriminate between quinoa samples. 

Following this data processing strategy, a total of 49 proteins were detected across the different quinoa samples, including the four varieties, the two raw materials (seeds and grains), the two farming conditions (conventional and organic), and the two processing methods (boiling and extrusion). To tentatively identify these proteins based on their M_r_, we compared them with the experimental proteome map of the Salcedo samples obtained in a separate study by LC-MS/MS shotgun proteomics [47]. Table 1 lists the experimental M_r_ calculated for the detected proteins, their theoretical M_r_, the accession number (ID), and the names of the 31 tentatively identified proteins out of the 49 detected proteins. Note that in many cases, several possible identifications were provided because the mass accuracy and resolution of the full-scan MALDI-TOF mass spectrometer was not enough for an unequivocal identification.

### 3.2. Multivariate Data Analysis

#### 3.2.1. Discrimination of Conventional and Organic Quinoa

Multivariate data analysis was carried out, considering the intensities of the 49 detected proteins in the different protein extracts. To simplify data interpretation for differentiating conventional and organic quinoa samples, only the protein fingerprints from the 48 raw samples corresponding to seeds and grains grown under both farming conditions were considered. Initially, unsupervised PCA was employed to visualize trends and identify outliers from the scores plot (Supplementary Figure S1) [28,31]. Two principal components (PCs) explained a total variance of 52.9% (Supplementary Figure S1). Given the absence of distinct trends in the scores plot across samples from the four different white quinoa varieties, even when increasing the number of components, the representation of the samples was solely based on farming conditions. As can be observed, PC1 (33.9% of the explained variance) revealed differential clustering between conventional and organic quinoa samples, while PC2 (19.0% of the explained variance) separated samples within these two groups. Additionally, only two samples corresponding to the V2 quinoa variety from conventional farming appeared outside the 95% confidence ellipse of the scores plot and were identified as outliers, thus excluding them from the supervised PLS-DA analysis. 

**Figure 3:**
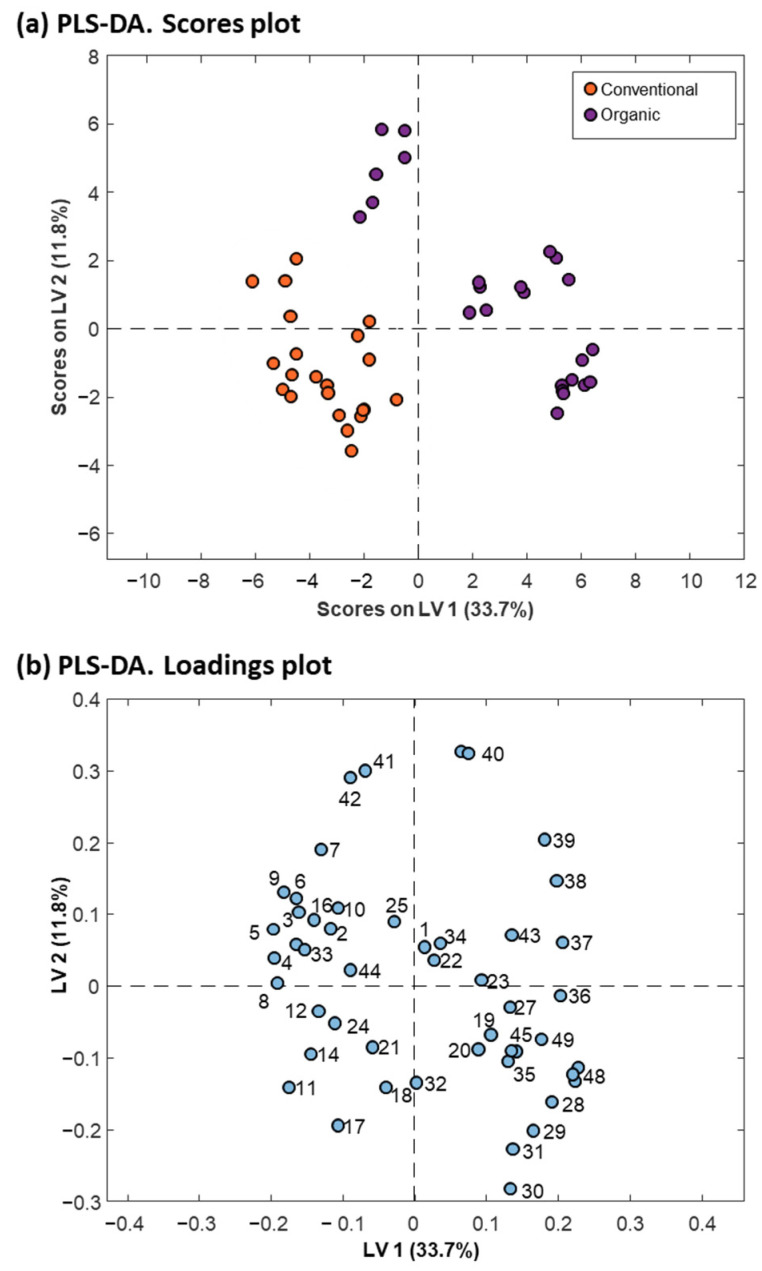
PLS−DA scores plot (**a**) and loadings plot (**b**) derived from the analysis of 46 protein extracts from conventional and organic raw quinoa varieties (seed and grain) using the intensities of the 49 protein peaks detected by MALDIquant.

**Figure 4:**
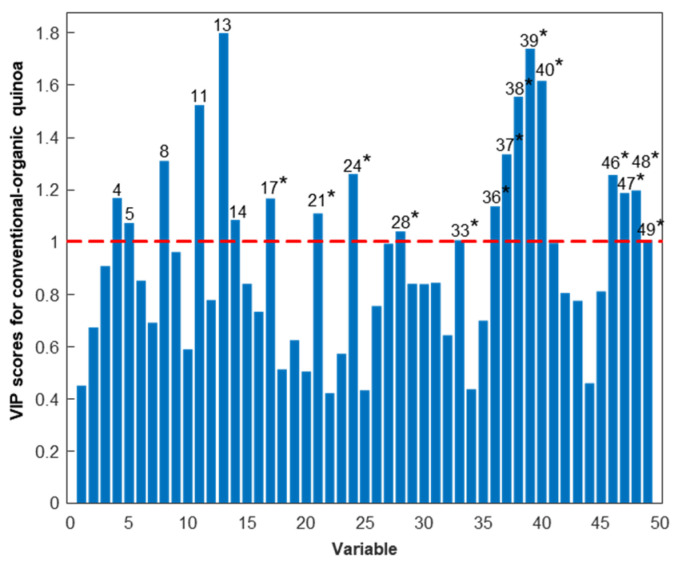
VIP scores of the different protein variables when considering the separation between conventional and organic raw quinoa sample classes. Protein variables with a VIP score greater than one are numbered, and those tentatively identified are marked with an asterisk (*) (as in Table 1).

A PLS-DA model considering two classes was established to enhance discrimination and identify the protein variables significantly contributing to the differentiation between quinoa farming conditions. The scores plot of the PLS-DA model, with two latent variables (LVs), accounting for 45.5% of the explained variance and illustrated in Figure 3a, effectively demonstrated discrimination between conventional and organic quinoa samples, suggesting that farming conditions induced differences at the protein level [51]. The loadings plot depicted the contribution of the different protein variables to the LVs (Figure 3b), while the VIP scores provided additional information to reveal the relevant contribution of these variables for discrimination (Figure 4). As shown in Figure 4, 20 of the 49 detected proteins were found to be the most important for discriminating between conventional and organic quinoa (VIP > 1) [44]. The M_r_ values of this subset of 20 proteins ranged between 5000 and 25,000. Additionally, 14 of these relevant proteins were tentatively identified, as summarized in Table 1. Notably, several of the tentatively identified proteins ranked at the top of VIP values (VIP > 1.5), including protein 38 (VIP value of 1.56), protein 39 (VIP value of 1.74), and protein 40 (VIP value of 1.62), which emerged as primary discriminants between conventional and organic quinoa (see Table 1 for the identities). Overall, these 20 proteins, selected based on their discriminatory potential, could be considered critical markers for discriminating quinoa grown under varying agroecological conditions.

#### 3.2.2. Discrimination of Raw and Processed Quinoa 

**Figure 5:**
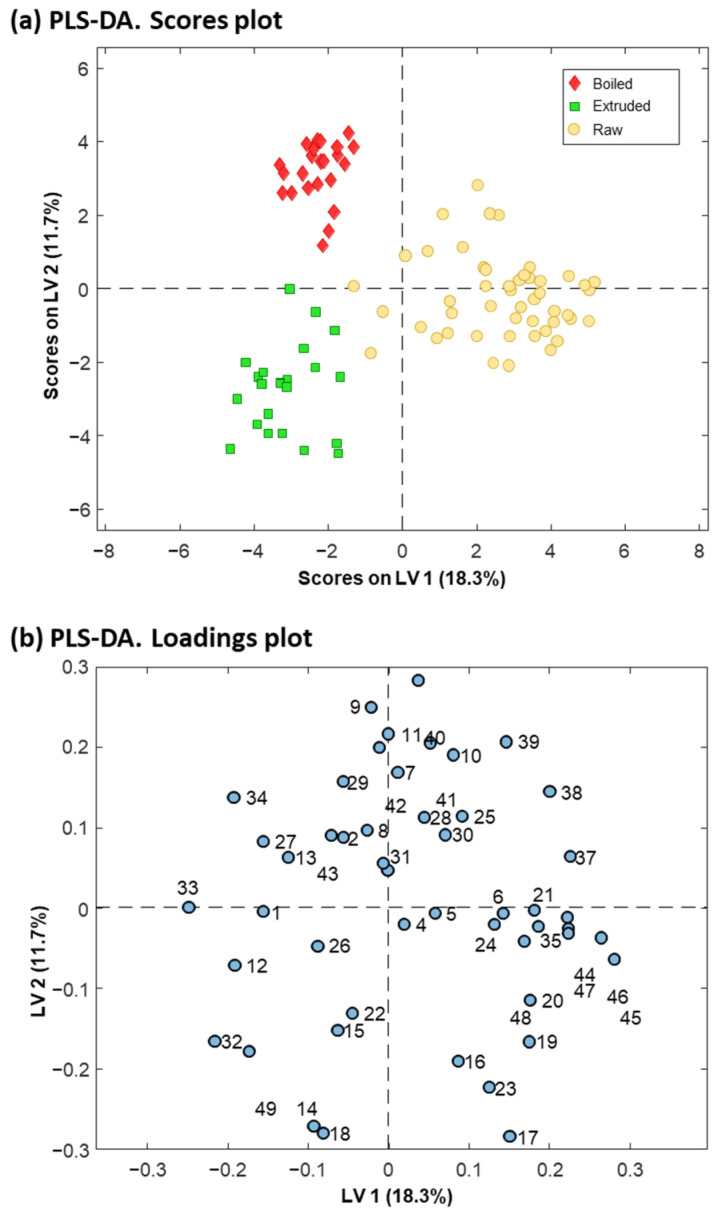
PLS−DA scores plot (**a**) and loadings plot (**b**) derived from the analysis of 96 protein extracts from seed, grain, boiled, and extruded quinoa varieties from conventional and organic farming using the intensities of the 49 protein peaks detected by MALDIquant.

In order to differentiate between raw and processed quinoa, a PCA was conducted considering the intensities of the 49 detected proteins in the different protein extracts of seed, grain, boiled, and extruded quinoa samples from conventional and organic farming. As can be observed in the scores plot of Supplementary Figure S2, two PCs explained a total variance of 38.3%. To focus on the impact of boiling and extrusion on raw quinoa proteins and clarify the evaluation of sample trends and clustering, samples were represented in the score plot without considering the different varieties and farming conditions. PC2 (16.4% of the explained variance) facilitated the separation of boiled and extruded quinoa from grain and seed samples, predominantly located along the positive axis of PC2. Furthermore, PC1 (21.9% of the explained variance) led to a very slight separation between boiled and extruded quinoa samples, while grain and seed samples were distributed and overlapped along the axis of this component. Since no clear clustering was observed for seed and grain samples, we decided to consider raw quinoa samples as a single class for subsequent PLS-DA analysis (Figure 5).

**Figure 6:**
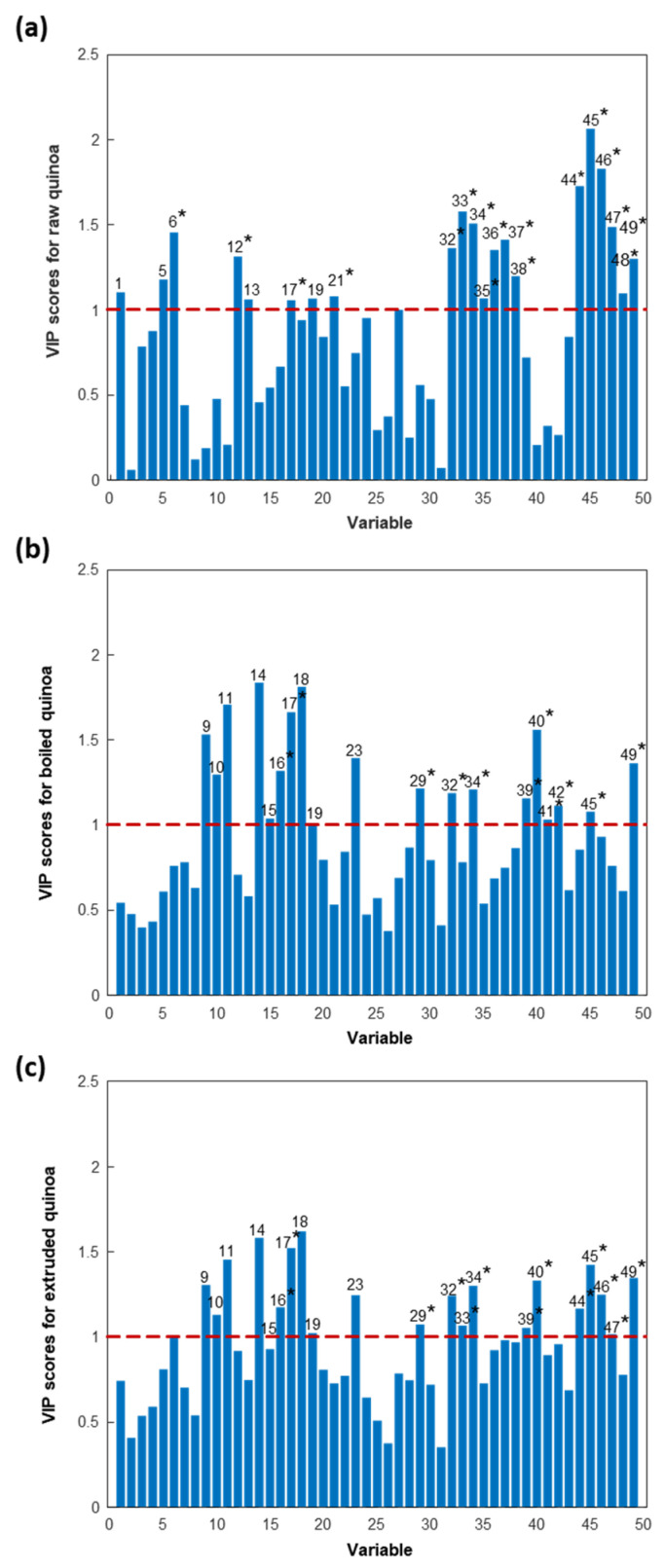
VIP scores of the different protein variables when considering the separation of (**a**) raw (seed and grain), (**b**) boiled, and (**c**) extruded quinoa sample classes from the other two sample classes. Protein variables with a VIP score greater than one are numbered, and those tentatively identified are marked with an asterisk (*) (as in Table 1).

A PLS-DA model considering three classes (i.e., raw (seed and grain), boiled, and extruded quinoa samples) was established for improved discrimination between raw and processed quinoa samples, as well as to identify the most relevant variables for the discrimination. Figure 5a displays the scores plot of the PLS-DA model with two LVs (accounting for 30% of the explained variance), revealing a complete separation with a clear division between boiled, extruded, and raw quinoa samples. This suggested that quinoa processing affected raw quinoa proteins, as well as demonstrating a differential effect of boiling and extrusion. VIP values (Figure 6) were calculated to assess the level of contribution of the different protein variables represented in the loadings plot of Figure 5b for the discrimination of the three classes of quinoa samples. 

Figure 6 shows, in each case, the VIP plots for the discrimination of raw, boiled, and extruded quinoa samples from the other two sample classes. Analysing Table 1 and Figure 6, it can be concluded that 38 of the 49 detected proteins were significant for the discrimination of quinoa sample classes (VIP > 1), constituting critical markers of quinoa processing. It is important to note that this protein set included the 20 proteins necessary to distinguish between farming practices. Additionally, 25 of the 38 relevant proteins were tentatively identified (5000 < M_r_ < 25,000), as summarized in Table 1 and marked with an asterisk in Figure 6. Notably, several of the tentatively identified proteins exhibited high VIP values (VIP > 1.5), underscoring their significance in distinguishing between quinoa processing methods. Specifically, proteins 33 (VIP value of 1.58), 44 (VIP value of 1.72), 45 (VIP value of 2.06), and 46 (VIP value of 1.83) emerged as primary discriminants between raw and boiled/extruded quinoa. Similarly, proteins 17 (VIP value of 1.66) and 40 (VIP value of 1.56) were pivotal in discriminating between boiled and raw/extruded quinoa, while protein 17 (VIP value of 1.52) also played a crucial role in differentiating extruded and raw/boiled quinoa.

## 4. Conclusions

In this study, we presented a rapid and simple chemometrics-assisted MALDI-TOF-MS method to assess the influence of conventional and organic farming, boiling, and extrusion on protein profiles across various white quinoa grain varieties. Once the raw mass spectra had been acquired appropriately, we employed MALDIquant for data processing, enabling the resolution of complexities within the mass spectra and the reliable detection of proteins. A total of 49 proteins were detected, with 31 tentatively identified. The global fingerprints, comprising the intensity values of these proteins, were subsequently subjected to multivariate data analysis. Our results revealed a PLS-DA model for distinguishing between conventional and organic farming samples, with 20 out of the 49 detected proteins proving critical for differentiation (14 of which were identified). These 20 proteins were also relevant for discriminating between raw and processed samples, which required a total of 38 proteins for an effective differentiation by PLS-DA (25 of which were identified). This global profiling approach allows protein fingerprinting and chemometrics analysis to evaluate differences at the protein level in quinoa grains, facilitating the assessment of farming practices and quality changes during food processing. Further research will be needed to assess the impact of these differences at the nutritional and immunonutritional levels. Additionally, the potential application of the presented approach extends to other areas of food analysis, especially when dealing with complex mass spectra with highly overlapped peaks. 

## Figures and Tables

**Table 1:** List of proteins detected by MALDI-TOF-MS used as variables for multivariate data analysis with their corresponding experimental M_r_, PLS-DA VIP score values for discrimination between farming and processing conditions, and tentative identifications. Theoretical M_r_, accession number (ID), and protein name were based on an experimental proteome map of the Salcedo samples obtained in a separate study by LC-MS/MS shotgun proteomics [47].

Multivariate Data Analysis Protein Variables ^a^							Tentative Identifications ^b^	
Protein	Experimental M_r_ ^c^	PLS-DA VIP Scores ^d^					Theoretical M_r_	Accession Number (ID) ^e^ and Protein Name
		Farming		Processing				
		C_raw_ ^f^	O_raw_ ^f^	Raw	Boiled	Extruded		
1	4231	0.45	0.45	**1.10**	0.54	0.74		-
2	4445	0.67	0.67	0.06	0.48	0.41		-
3	4650	0.91	0.91	0.78	0.40	0.53		-
4	4951	**1.17**	**1.17**	0.87	0.43	0.59		-
5	5180	**1.07**	**1.07**	**1.18**	0.61	0.81		-
6	5304	0.85	0.85	1.45 *	0.76	1.00	5307	XP_021736943.1 wound-induced basic protein-like
7	5460	0.69	0.69	0.44	0.78	0.70		-
8	5587	**1.31**	**1.31**	0.12	0.63	0.54		-
9	5767	0.96	0.96	0.19	**1.53**	**1.30**		-
10	5934	0.59	0.59	0.47	**1.29**	**1.13**		-
11	6184	**1.52**	**1.52**	0.20	**1.70**	**1.45**		-
12	6391	0.78	0.78	1.31 *	0.70	0.91	6413	XP_021764391.1 40S ribosomal protein S29
13	6818	**1.80**	**1.80**	**1.06**	0.58	0.75		-
14	7063	**1.08**	**1.08**	0.46	**1.84**	**1.58**		-
15	7435	0.84	0.84	0.54	**1.04**	0.93		
16	7730	0.73	0.73	0.67	1.32 *	1.17 *	7747	XP_021714409.1 uncharacterized protein LOC110682385
17	7983	1.17 *	1.17 *	1.06 *	1.66 *	1.52 *	7974	XP_021773921.1 metallothionein-like protein 4B
18	8221	0.51	0.51	0.94	**1.81**	**1.62**		-
19	8465	0.62	0.62	**1.06**	**1.01**	**1.02**		-
20	8635	0.50	0.50	0.84	0.79	0.81		-
21	8840	1.11 *	1.11 *	1.08 *	0.53	0.73	8806	XP_021718956.1 protein DELETION OF SUV3 SUPPRESSOR 1(I)-like
							8814	XP_021729007.1 uncharacterized protein LOC110696048
							8823	XP_021762602.1 defensin-like protein
22	9054	0.42	0.42	0.55	0.84	0.77	9076	XP_021768105.1 late seed maturation protein P8B6-like
23	9314	0.57	0.57	0.74	**1.39**	**1.24**		-
24	9695	1.26 *	1.26 *	0.95	0.47	0.64	9692	XP_021747091.1 sm-like protein LSM5
25	10,106	0.43	0.43	0.29	0.57	0.51		-
26	10,703	0.75	0.75	0.37	0.37	0.37	10,689	XP_021714810.1 uncharacterized protein LOC110682782
							10,724	XP_021756490.1 sm-like protein LSM8
							10,736	XP_021756753.1 60S ribosomal protein L37-3
							10,750	XP_021768220.1 sm-like protein LSM7
27	10,947	0.99	0.99	1.00	0.69	0.79	10,920	XP_021761138.1 mitochondrial import inner membrane translocase subunit Tim9
							10,928	XP_021746329.1 probable steroid-binding protein 3
							10,989	XP_021761862.1 peamaclein-like
28	11,274	1.04 *	1.04 *	0.25	0.86	0.75	11,270	XP_021771595.1 sm-like protein LSM3A
							11,308	XP_021716413.1 60S acidic ribosomal protein P2-4-like
29	11,489	0.84	0.84	0.56	1.21 *	1.07 *	11,449	XP_021727941.1 NADH dehydrogenase
							11,458	XP_021766637.1 non-specific lipid-transfer protein-like
							11,517	XP_021754863.1 thioredoxin M-type, chloroplastic-like isoform X2
30	11,779	0.84	0.84	0.47	0.79	0.72	11,723	XP_021772578.1 RNA polymerase II transcriptional coactivator KIWI-like isoform X1
							11,772	XP_021755694.1 uncharacterized protein LOC110720913
							11,797	XP_021763483.1 small ubiquitin-related modifier 1-like
31	12,043	0.84	0.84	0.07	0.41	0.35	11,992	YP_009380236.1 ribosomal protein S18 (chloroplast)
							12,050	XP_021776279.1 peptidyl-prolyl cis-trans isomerase FKBP12-like
							12,055	XP_021765385.1 NADH dehydrogenase
							12,064	XP_021774292.1 huntingtin-interacting protein K-like
32	12,362	0.64	0.64	1.36 *	1.19 *	1.24 *	12,301	XP_021760438.1 gibberellin-regulated protein 9-like
							12,315	XP_021716755.1 uncharacterized protein At2g27730, mitochondrial-like
							12,332	XP_021765334.1 V-type proton ATPase subunit G 1-like
							12,375	XP_021720641.1 60S ribosomal protein L30
							12,407	XP_021761775.1 uncharacterized protein LOC110726608
							12,413	XP_021773050.1 60S ribosomal protein L36-2-like
							12,420	XP_021738644.1 40S ribosomal protein S25-like
33	12,801	1.01 *	1.01 *	1.58 *	0.78	1.06 *	12,827	XP_021769120.1 60S ribosomal protein L35a-3
							12,849	XP_021757241.1 nodulin-related protein 1-like
							12,855	XP_021718430.1 nodulin-related protein 1-like
34	13,220	0.43	0.43	1.50 *	1.21 *	1.30 *	13,205	XP_021758336.1 thioredoxin H-type 1-like
							13,231	XP_021759897.1 thioredoxin H-type 1-like
35	16,215	0.70	0.70	1.06 *	0.54	0.72	16,134	XP_021716351.1 ferredoxin, root R-B2-like
							16,149	XP_021747601.1 uncharacterized protein LOC110713466
							16,165	XP_021730244.1 outer envelope pore protein 16-2, chloroplastic-like isoform X2
							16,200	XP_021717733.1 high mobility group B protein 3-like
							16,215	XP_021716749.1 ferredoxin, root R-B2-like
							16,216	XP_021754488.1 high mobility group B protein 3-like
							16,239	XP_021762815.1 uncharacterized protein At5g48480-like
							16,250	XP_021766528.1 40S ribosomal protein S14-2
							16,289	XP_021721762.1 oleosin 1-like
36	16,514	1.13 *	1.13 *	1.35 *	0.68	0.92	16,458	XP_021733518.1 uncharacterized protein At5g48480-like
							16,464	XP_021761922.1 uncharacterized protein LOC110726743
							16,469	XP_021746531.1 60S ribosomal protein L27a-3-like
							16,474	XP_021769235.1 glycine cleavage system H protein 2, mitochondrial-like
							16,524	XP_021768671.1 60S ribosomal protein L27a-3-like
							16,568	XP_021762909.1 uncharacterized protein LOC110727639
							16,570	XP_021732568.1 uncharacterized protein LOC110699354
37	16,693	1.34 *	1.34 *	1.41 *	0.75	0.98	16,616	XP_021755504.1 2S albumin-like
							16,624	XP_021751394.1 60S ribosomal protein L26-1
							16,625	XP_021730224.1 probable calcium-binding protein CML13
							16,636	XP_021760375.1 eukaryotic translation initiation factor 1A
							16,651	XP_021731588.1 glycine-rich RNA-binding, abscisic acid-inducible protein-like
							16,685	XP_021735190.1 ubiquitin-conjugating enzyme E2 variant 1D-like
							16,693	XP_021774210.1 60S ribosomal protein L28-1-like
							16,702	XP_021717270.1 blue copper protein-like isoform X2
							16,742	XP_021720407.1 17.4 kDa class III heat shock protein-like
							16,758	XP_021766054.1 uncharacterized protein LOC110730552
38	16,897	1.56 *	1.56 *	1.20 *	0.86	0.97	16,833	XP_021733717.1 40S ribosomal protein S16-like
							16,834	XP_021776507.1 calmodulin-7-like
							16,860	XP_021754554.1 calmodulin
							16,877	XP_021749775.1 peptidyl-prolyl cis-trans isomerase FKBP15-1-like
							16,884	XP_021716580.1 17.4 kDa class III heat shock protein-like
							16,933	XP_021735458.1 probable prefoldin subunit 5
							16,942	XP_021731073.1 thiosulfate sulfurtransferase 16, chloroplastic-like isoform X2
							16,946	XP_021743153.1 uncharacterized protein LOC110709246
							16,962	XP_021758167.1 transcription initiation factor TFIID subunit 15b-like
39	17,101	1.74 *	1.74 *	0.72	1.16 *	1.05 *	17,026	XP_021751891.1 NADH dehydrogenase
							17,040	XP_021771944.1 DNA-directed RNA polymerases II, IV and V subunit 8B-like
							17,048	XP_021739940.1 uncharacterized protein LOC110706342
							17,111	XP_021765383.1 40S ribosomal protein S13-like
							17,129	XP_021766190.1 uncharacterized protein LOC110730679
							17,131	XP_021740721.1 MLP-like protein 423
							17,143	XP_021769150.1 17.8 kDa class I heat shock protein-like
40	17,326	1.62 *	1.62 *	0.20	1.56 *	1.33 *	17,290	XP_021770408.1 outer envelope pore protein 16-3, chloroplastic/mitochondrial-like
							17,301	XP_021729636.1 NADH dehydrogenase
							17,330	XP_021717756.1 uncharacterized protein LOC110685525
							17,340	XP_021747441.1 eukaryotic translation initiation factor 5A-4-like
							17,350	XP_021764293.1 40S ribosomal protein S15-4-like
							17,355	YP_009380273.1 ribosomal protein S7 (chloroplast)
							17,366	XP_021747435.1 eukaryotic translation initiation factor 5A-like
							17,376	XP_021720177.1 ubiquitin-NEDD8-like protein RUB2
							17,385	XP_021748235.1 60S ribosomal protein L23A
41	17,617	**1.00** *	**1.00** *	0.32	1.03 *	0.89	17,532	XP_021765685.1 glycine cleavage system H protein, mitochondrial
							17,543	XP_021768154.1 glycine cleavage system H protein, mitochondrial-like
							17,560	XP_021731505.1 oleosin 1-like
							17,562	XP_021736891.1 peroxiredoxin-2B-like
							17,572	XP_021732018.1 peroxiredoxin-2B-like
							17,592	XP_021756471.1 putative 4-hydroxy-4-methyl-2-oxoglutarate aldolase 3
							17,604	XP_021735589.1 nascent polypeptide-associated complex subunit beta-like
							17,622	XP_021733122.1 protein mago nashi homolog 2
							17,652	XP_021743932.1 histidine-containing phosphotransfer protein 1-like
							17,665	XP_021753630.1 uncharacterized protein LOC110719020
							17,675	XP_021759953.1 nascent polypeptide-associated complex subunit beta-like
							17,700	XP_021745442.1 40S ribosomal protein S11-3
42	17,879	0.80	0.80	0.26	1.11 *	0.96	17,803	XP_021769395.1 40S ribosomal protein S11-like
							17,855	XP_021773311.1 60S ribosomal protein L12-1
							17,916	XP_021748317.1 desiccation protectant protein Lea14 homolog
							17,939	XP_021749487.1 MLP-like protein 43
							17,969	XP_021715429.1 universal stress protein PHOS34-like
43	18,311	0.77	0.77	0.84	0.62	0.69	18,221	XP_021738830.1 oleosin 16 kDa
							18,224	XP_021737967.1 MFP1 attachment factor 1-like
							18,238	XP_021765145.1 60S ribosomal protein L24-like
							18,240	XP_021753128.1 peptidyl-prolyl cis-trans isomerase 1-like
							18,252	XP_021763237.1 pathogenesis-related protein STH-21-like
							18,254	XP_021775867.1 peptidyl-prolyl cis-trans isomerase 1
							18,258	XP_021769094.1 18.3 kDa class I heat shock protein-like
							18,271	XP_021730326.1 universal stress protein PHOS32
							18,276	XP_021744114.1 17.3 kDa class II heat shock protein-like
							18,317	XP_021752091.1 probable NADH dehydrogenase
							18,348	XP_021738936.1 17.3 kDa class II heat shock protein-like
							18,348	XP_021732306.1 pathogenesis-related protein STH-21-like
							18,348	XP_021725562.1 deoxyuridine 5-triphosphate nucleotidohydrolase
							18,366	XP_021774711.1 50S ribosomal protein L18, chloroplastic
44	20,349	0.46	0.46	1.72 *	0.85	1.16 *	20,301	XP_021763546.1 30S ribosomal protein 3, chloroplastic
							20,406	XP_021734303.1 HMG-Y-related protein A-like
45	20,556	0.81	0.81	2.06 *	1.08 *	1.42 *	20,466	XP_021727144.1 21 kDa seed protein-like
							20,499	XP_021763320.1 photosystem II reaction center Psb28 protein-like
							20,522	XP_021744010.1 succinate dehydrogenase assembly factor 2, mitochondrial-like
							20,523	XP_021729294.1 uncharacterized protein LOC110696308
							20,557	XP_021766022.1 PLAT domain-containing protein 3-like
							20,565	XP_021741243.1 putative H/ACA ribonucleoprotein complex subunit 1-like protein 1
							20,592	XP_021769990.1 ADP-ribosylation factor 1-like
							20,619	XP_021752903.1 thioredoxin-like protein CITRX, chloroplastic
46	20,780	1.25 *	1.25 *	1.83 *	0.93	1.25 *	20,736	XP_021773813.1 adenylate kinase isoenzyme 6 homolog
							20,739	XP_021740322.1 protein CutA, chloroplastic-like
							20,778	XP_021761077.1 peroxiredoxin-2F, mitochondrial-like isoform X1
							20,799	XP_021753718.1 60S ribosomal protein L11-1
							20,801	XP_021772257.1 HMG-Y-related protein A-like
							20,844	XP_021763208.1 60S ribosomal protein L18-3-like
							20,848	XP_021738998.1 protein OPI10 homolog
							20,852	XP_021763370.1 monothiol glutaredoxin-S10-like
47	21,075	1.19 *	1.19 *	1.48 *	0.76	1.01 *	21,027	XP_021750037.1 uncharacterized protein LOC110715738
							21,031	XP_021730777.1 thioredoxin O2, mitochondrial-like isoform X2
							21,055	XP_021766443.1 lactoylglutathione lyase isoform X2
							21,077	XP_021756715.1 uncharacterized protein LOC110721825
							21,107	XP_021727997.1 50S ribosomal protein L27, chloroplastic
							21,121	XP_021733985.1 glycine-rich RNA-binding protein 3, mitochondrial-like
							21,149	XP_021736893.1 probable inactive nicotinamidase At3g16190
							21,170	XP_021763161.1 uncharacterized protein Os08g0359500-like
							21,172	XP_021732021.1 probable inactive nicotinamidase At3g16190
48	21,343	1.20 *	1.20 *	1.09 *	0.61	0.78	21,241	XP_021720070.1 ankyrin repeat and SAM domain-containing protein 6-like isoform X2
							21,268	XP_021771518.1 uncharacterized protein LOC110735639
							21,294	XP_021764214.1 cyclic phosphodiesterase-like
							21,326	XP_021763910.1 60S ribosomal protein L18a-2
							21,364	XP_021754795.1 50S ribosomal protein L24, chloroplastic-like
							21,376	XP_021718085.1 60S ribosomal protein L18a
							21,433	XP_021730369.1 probable prefoldin subunit 3
49	22,079	1.01 *	1.01 *	1.30 *	**1.36** ^*^	1.34 *	21,984	XP_021772119.1 RNA-binding protein Y14-like
							22,018	XP_021763572.1 40S ribosomal protein S7-like
							22,034	XP_021761714.1 histone H1-like
							22,040	YP_009380239.1 ClpP (chloroplast)
							22,088	XP_021766393.1 50S ribosomal protein L9, chloroplastic-like

^a^ PLS−DA variables correspond to the protein peaks detected by MALDIquant. ^b^ The experimental proteome map of the same samples obtained in a separate study by LC-MS/MS shotgun proteomics [47] was used as a reference for the tentative identification. A mass error ±0.5% between the theoretical and experimental M_r_ was considered acceptable for proposing an identity. This threshold value was established considering the mass error observed for the analysis of a ribonuclease A standard (from a bovine pancreas) under the same instrumental conditions, M_r_ = 13,690). ^c^ Experimental M_r_ were calculated from the *m*/*z* values considering the formation of single-charged molecular ions by MALDI-TOF-MS. ^d^ VIP scores > 1 were considered important for discrimination and are marked in bold, and tentatively identified scores were marked with an asterisk (*). ^e^ Accession numbers (IDs) of the identified proteins correspond to the IDs of the indicated LC-MS/MS shotgun proteomics work [47]. The tentatively identified proteins fulfilling the acceptance criterium are ordered by the Andromeda score values obtained by LC-MS/MS, which are a measure of the reliability of their identification. ^f^ C_raw_ and O_raw_ quinoa correspond to raw (seeds and grains) quinoa from conventional and organic farming, respectively.

## Data Availability

The original contributions presented in the study are included in the article/Supplementary Materials, further inquiries can be directed to the corresponding author.

## Supplementary Materials

The following supporting information can be downloaded at: https://www.mdpi.com/article/10.3390/foods13121906/s1, Supplementary Figure S1. PCA scores plot derived from the analysis of 48 protein extracts from conventional and organic raw quinoa varieties (seed and grain) using the intensities of the 49 protein peaks detected by MALDIquant.; Supplementary Figure S2. PCA scores plot derived from the analysis of 96 protein extracts from seed, grain, boiled, and extruded quinoa varieties from conventional and organic farming using the intensities of the 49 protein peaks detected by MALDIquant.

## References

[1] Aloisi I., Parrotta L., Ruiz K.B., Landi C., Bini L., Cai G., Biondi S., Del Duca S. (2016). New Insight into Quinoa Seed Quality under Salinity: Changes in Proteomic and Amino Acid Profiles, Phenolic Content, and Antioxidant Activity of Protein Extracts. Front. Plant Sci..

[2] Mu H., Xue S., Sun Q., Shi J., Zhang D., Wang D., Wei J. (2023). Research Progress of Quinoa Seeds (*Chenopodium quinoa* Wild.): Nutritional Components, Technological Treatment, and Application. Foods.

[3] Chaudhary N., Walia S., Kumar R. (2023). Functional Composition, Physiological Effect and Agronomy of Future Food Quinoa (Chenopodium Quinoa Willd.): A Review. J. Food Compos. Anal..

[4] Angeli V., Silva P.M., Massuela D.C., Khan M.W., Hamar A., Khajehei F., Graeff-Hönninger S., Piatti C. (2020). Quinoa (*Chenopodium quinoa* Willd.): An Overview of the Potentials of the “Golden Grain” and Socio-Economic and Environmental Aspects of Its Cultivation and Marketization. Foods.

[5] Niro S., D’Agostino A., Fratianni A., Cinquanta L., Panfili G. (2019). Gluten-Free Alternative Grains: Nutritional Evaluation and Bioactive Compounds. Foods.

[6] Mhada M., Metougui M.L., El Hazzam K., El Kacimi K., Yasri A. (2020). Variations of Saponins, Minerals and Total Phenolic Compounds Due to Processing and Cooking of Quinoa (*Chenopodium quinoa* Willd.) Seeds. Foods.

[7] Hussain M.I., Farooq M., Syed Q.A., Ishaq A., Al-Ghamdi A.A., Hatamleh A.A. (2021). Botany, Nutritional Value, Phytochemical Composition and Biological Activities of Quinoa. Plants.

[8] Ceyhun Sezgin A., Sanlier N. (2019). A New Generation Plant for the Conventional Cuisine: Quinoa (*Chenopodium quinoa* Willd.). Trends Food Sci. Technol..

[9] El-Serafy R.S., El-Sheshtawy A.-N.A., Abd El-Razek U.A., Abd El-Hakim A.F., Hasham M.M.A., Sami R., Khojah E., Al-Mushhin A.A.M. (2021). Growth, Yield, Quality, and Phytochemical Behavior of Three Cultivars of Quinoa in Response to Moringa and Azolla Extracts under Organic Farming Conditions. Agronomy.

[10] Gomiero T. (2018). Food Quality Assessment in Organic vs. Conventional Agricultural Produce: Findings and Issues. Appl. Soil Ecol..

[11] Cancino-Espinoza E., Vázquez-Rowe I., Quispe I. (2018). Organic Quinoa (*Chenopodium quinoa* L.) Production in Peru: Environmental Hotspots and Food Security Considerations Using Life Cycle Assessment. Sci. Total Environ..

[12] Bazile D., Bertero D., Nieto C., FAO, CIRAD (2015). State of the Art Report on Quinoa around the World in 2013.

[13] Huang R., Huang K., Guan X., Li S., Cao H., Zhang Y., Lao X., Bao Y., Wang J. (2021). Effect of Defatting and Extruding Treatment on the Physicochemical and Storage Properties of Quinoa (*Chenopodium quinoa* Wild) Flour. LWT.

[14] Naozuka J., Oliveira P.V. (2012). Cooking Effects on Iron and Proteins Content of Beans (*Phaseolus vulgaris* L.) by GF AAS and MALDI-TOF MS. J. Braz. Chem. Soc..

[15] Scanlin L., Lewis K.A., Nadathur S.R., Wanasundara J.P.D., Scanlin L. (2017). Quinoa as a Sustainable Protein Source: Production, Nutrition, and Processing. Sustainable Protein Sources.

[16] Poza-Viejo L., Redondo-Nieto M., Matías J., Granado-Rodríguez S., Maestro-Gaitán I., Cruz V., Olmos E., Bolaños L., Reguera M. (2023). Shotgun Proteomics of Quinoa Seeds Reveals Chitinases Enrichment under Rainfed Conditions. Sci. Rep..

[17] Di Silvestre D., Passignani G., Rossi R., Ciuffo M., Turina M., Vigani G., Mauri P.L. (2022). Presence of a Mitovirus Is Associated with Alteration of the Mitochondrial Proteome, as Revealed by Protein–Protein Interaction (PPI) and Co-Expression Network Models in Chenopodium Quinoa Plants. Biology.

[18] Derbali W., Manaa A., Spengler B., Goussi R., Abideen Z., Ghezellou P., Abdelly C., Forreiter C., Koyro H.W. (2021). Comparative Proteomic Approach to Study the Salinity Effect on the Growth of Two Contrasting Quinoa Genotypes. Plant Physiol. Biochem..

[19] Rasouli F., Kiani-Pouya A., Shabala L., Li L., Tahir A., Yu M., Hedrich R., Chen Z., Wilson R., Zhang H. (2021). Salinity Effects on Guard Cell Proteome in Chenopodium Quinoa. Int. J. Mol. Sci..

[20] Wang Z., Wu Q., Kamruzzaman M. (2022). Portable NIR Spectroscopy and PLS Based Variable Selection for Adulteration Detection in Quinoa Flour. Food Control.

[21] Shotts M.L., Plans Pujolras M., Rossell C., Rodriguez-Saona L. (2018). Authentication of Indigenous Flours (Quinoa, Amaranth and Kañiwa) from the Andean Region Using a Portable ATR-Infrared Device in Combination with Pattern Recognition Analysis. J. Cereal Sci..

[22] Rodríguez S.D., Rolandelli G., Buera M.P. (2019). Detection of Quinoa Flour Adulteration by Means of FT-MIR Spectroscopy Combined with Chemometric Methods. Food Chem..

[23] Xue S.S., Tan J., Xie J.Y., Li M.F. (2021). Rapid, Simultaneous and Non-Destructive Determination of Maize Flour and Soybean Flour Adulterated in Quinoa Flour by Front-Face Synchronous Fluorescence Spectroscopy. Food Control.

[24] Kelis Cardoso V.G., Poppi R.J. (2021). Cleaner and Faster Method to Detect Adulteration in Cassava Starch Using Raman Spectroscopy and One-Class Support Vector Machine. Food Control.

[25] Ellis D.I., Brewster V.L., Dunn W.B., Allwood J.W., Golovanov A.P., Goodacre R. (2012). Fingerprinting Food: Current Technologies for the Detection of Food Adulteration and Contamination. Chem. Soc. Rev..

[26] Bansal S., Singh A., Mangal M., Mangal A.K., Kumar S. (2017). Food Adulteration: Sources, Health Risks, and Detection Methods. Crit. Rev. Food Sci. Nutr..

[27] Yang X., Xing B., Guo Y., Wang S., Guo H., Qin P., Hou C., Ren G. (2022). Rapid, Accurate and Simply-Operated Determination of Laboratory-Made Adulteration of Quinoa Flour with Rice Flour and Wheat Flour by Headspace Gas Chromatography-Ion Mobility Spectrometry. LWT.

[28] Galindo-Luján R., Pont L., Sanz-Nebot V., Benavente F. (2021). Classification of Quinoa Varieties Based on Protein Fingerprinting by Capillary Electrophoresis with Ultraviolet Absorption Diode Array Detection and Advanced Chemometrics. Food Chem..

[29] Galindo-Luján R., Caballero-Alcazar N., Pont L., Sanz-Nebot V., Benavente F. (2023). Fingerprinting of Quinoa Grain Protein Extracts by Liquid Chromatography with Spectrophotometric Detection for Chemometrics Discrimination. LWT.

[30] Galindo-Luján R., Pont L., Minic Z., Berezovski M.V., Sanz-Nebot V., Benavente F. (2021). Characterization and Differentiation of Quinoa Seed Proteomes by Label-Free Mass Spectrometry-Based Shotgun Proteomics. Food Chem..

[31] Galindo-Luján R., Pont L., Sanz-Nebot V., Benavente F. (2023). Protein Profiling and Classification of Commercial Quinoa Grains by MALDI-TOF-MS and Chemometrics. Food Chem..

[32] Galindo-Luján R., Pont L., Sanz-Nebot V., Benavente F. (2023). A Proteomics Data Mining Strategy for the Identification of Quinoa Grain Proteins with Potential Immunonutritional Bioactivities. Foods.

[33] R Development Core Team: R: A Language and Environment for Statistical Computing R Foundation for Statistical Computing. http://www.r-project.org/.

[34] Gibb S. MALDIquantForeign: Import/Export Routines for MALDIquant. 2014; pp. 1–7. https://cran.r-project.org/package=MALDIquantForeign.

[35] Gibb S., Strimmer K. (2017). Mass Spectrometry Analysis Using MALDIquant. Statistical Analysis of Proteomics, Metabolomics, and Lipidomics Data Using Mass Spectrometry.

[36] Purohit P.V., Rocke D.M. (2003). Discriminant Models for High-Throughput Proteomics Mass Spectrometer Data. Proteomics.

[37] Savitzky A., Golay M.J.E. (1964). Smoothing and Differentiation of Data by Simplified Least Squares Procedures. Anal. Chem..

[38] Ryan C.G., Clayton E., Griffin W.L., Sie S.H., Cousens D.R. (1988). SNIP, a Statistics-Sensitive Background Treatment for the Quantitative Analysis of PIXE Spectra in Geoscience Applications. Nucl. Instrum. Methods Phys. Res. B.

[39] Borgaonkar S.P., Hocker H., Shin H., Markey M.K. (2010). Comparison of Normalization Methods for the Identification of Biomarkers Using MALDI-TOF and SELDI-TOF Mass Spectra. OMICS.

[40] Cleveland W.S. (1979). Robust Locally Weighted Regression and Smoothing Scatterplots. J. Am. Stat. Assoc..

[41] Friedman J.H. (1984). A Variable Span Smoother. Laboratory for Computational Statistics, Stanford University Technical Report No. 5. J. Am. Stat. Assoc..

[42] Barker M., Rayens W. (2003). Partial Least Squares for Discrimination. J. Chemom..

[43] Ballabio D., Consonni V. (2013). Classification Tools in Chemistry. Part 1: Linear Models. PLS-DA. Anal. Methods.

[44] Wold S., Sjöström M., Eriksson L. (2001). PLS-Regression: A Basic Tool of Chemometrics. Chemom. Intell. Lab. Syst..

[45] Worley B., Halouska S., Powers R. (2013). Utilities for Quantifying Separation in PCA/PLS-DA Scores Plots. Anal. Biochem..

[46] Mehmood T., Liland K.H., Snipen L., Sæbø S. (2012). A Review of Variable Selection Methods in Partial Least Squares Regression. Chemom. Intell. Lab. Syst..

[47] Galindo-Luján R., Pont L., Minic Z., Berezovski M.V., Quispe F., Sanz-Nebot V., Benavente F. (2024). Comprehensive Characterization of Raw and Processed Quinoa from Conventional and Organic Farming by Label-Free Shotgun Proteomics. https://ssrn.com/abstract=4774018.

[48] Soladoye O.P., Juárez M.L., Aalhus J.L., Shand P., Estévez M. (2015). Protein Oxidation in Processed Meat: Mechanisms and Potential Implications on Human Health. Compr. Rev. Food Sci. Food Saf..

[49] Santé-Lhoutellier V., Astruc T., Marinova P., Greve E., Gatellier P. (2008). Effect of Meat Cooking on Physicochemical State and in Vitro Digestibility of Myofibrillar Proteins. J. Agric. Food Chem..

[50] Tang H., Fu T., Feng Y., Zhang S., Wang C., Zhang D. (2019). Effect of Heat Treatment on Solubility, Surface Hydrophobicity and Structure of Rice Bran Albumin and Globulin. Qual. Assur. Saf. Crops Foods.

[51] Xiao R., Li L., Ma Y. (2019). A Label-Free Proteomic Approach Differentiates between Conventional and Organic Rice. J. Food Compos. Anal..

