# Supplementary material for: Matrix-Assisted Laser Desorption Ionization Time-of-Flight Mass Spectrometry Combined with Chemometrics for Protein Profiling and Classification of Boiled and Extruded Quinoa from Conventional and Organic Crops
Source: Foods. 2024 Jun 17;13(12):1906. doi: 10.3390/foods13121906 (PMC11203106; doi:10.3390/foods13121906)
Supplement: Supplementary file 1 [file foods-13-01906-s001.zip › foods-3034446-supplementary.pdf]

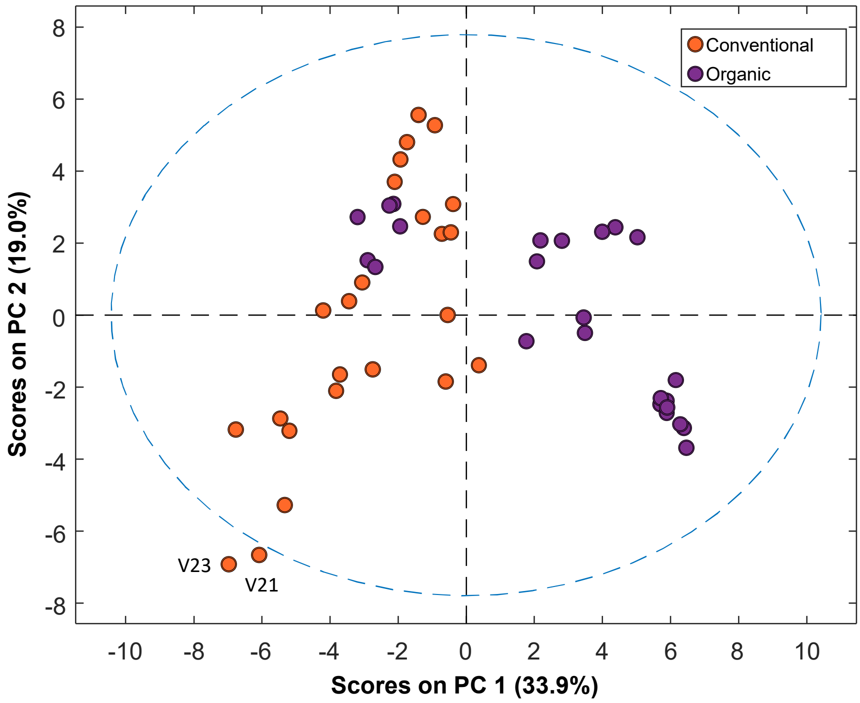

Supplementary Figure S1. PCA scores plot derived from the analysis of 48 protein extracts from conventional and organic raw quinoa varieties (seed and grain) using the intensities of the 49 protein peaks detected by MALDIquant. The two labeled samples from V2 variety (V2 = Santa Ana) were considered outliers and were discarded for PLS-DA analysis.

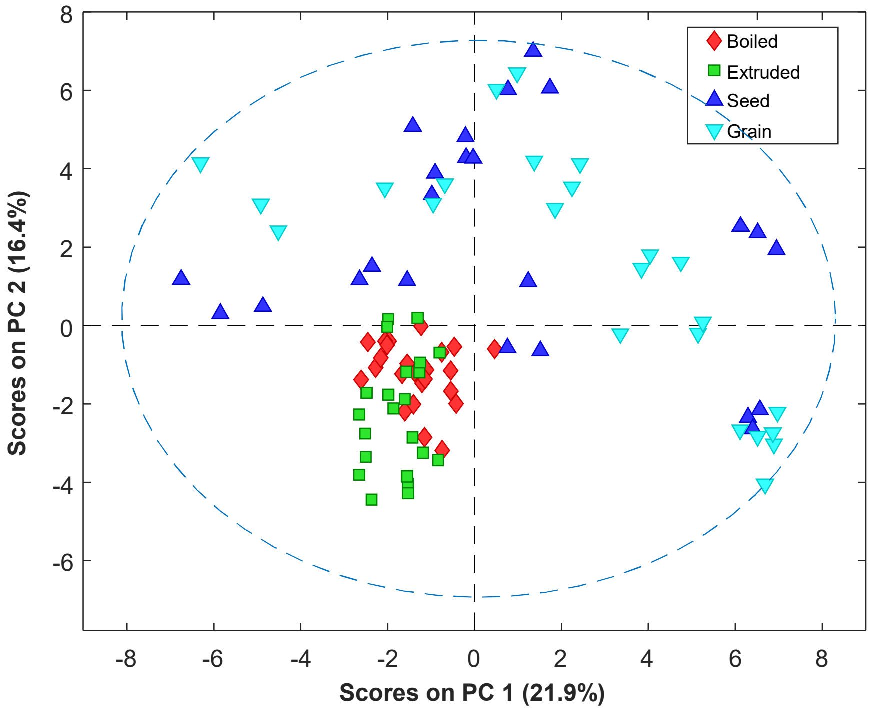

Supplementary Figure S2. PCA scores plot derived from the analysis of 96 protein extracts from seed, grain, boiled, and extruded quinoa varieties from conventional and organic farming using the intensities of the 49 protein peaks detected by MALDIquant. No outliers were detected.
